# Three new species and distributional records for *Paramaronius* Wittmer (Coleoptera, Cantharidae, Chauliognathinae)

**DOI:** 10.3897/zookeys.516.9529

**Published:** 2015-08-06

**Authors:** Gabriel Biffi

**Affiliations:** 1 Museu de Zoologia da Universidade de São Paulo, Av. Nazaré, 481 - Ipiranga, 04263-000, São Paulo, SP, Brazil

**Keywords:** Chauliognathini, description, distribution, key, taxonomy

## Abstract

Three new species of *Paramaronius* Wittmer from southeastern Brazil are described and illustrated: *Paramaronius serranus* **sp. n.**, *Paramaronius brancuccii* **sp. n.** and *Paramaronius cavipennis* **sp. n.** *Paramaronius impressipennis* (Pic) is redescribed, with supplementary description of the female. This species is recorded from Northeastern Brazil for the first time and its distribution pattern is discussed. A distribution map of *Paramaronius* in South America is provided. An identification key to all known species of the genus as well as photographs are given.

## Introduction

The genus *Paramaronius* Wittmer, 1963 is included in the group of short-elytra Chauliognathini and recognized by a remarkable sexual dimorphism (Wittmer 1963, Magis and Wittmer 1974, Brancucci 1981). It was established to include three species whose males present strong sculptural modifications and dense pubescense on the elytral surface: *Paramaronius kraatzi* (Pic, 1938), *Paramaronius freyi* Wittmer, 1963 and *Paramaronius murianus* Wittmer, 1963. Brancucci (1982) revised the genus, with description of *Paramaronius menieri* and proposed synonyms and new combinations: *Paramaronius impressipennis* (Pic, 1906) and *Paramaronius gounellei* (Pic, 1906) were transferred from *Maronius* Gorham, 1881, and *Paramaronius murianus* and *Maronius minasensis* Pic, 1934 considered junior synonyms of *Paramaronius gounellei*. Brancucci also presented an identification key and a phylogenetic hypothesis for the five species. Later, Brancucci (1983) described *Paramaronius campbelli*.

In addition to the elytral modifications, Brancucci (1982) proposed the reduced right paramere divided apically, and the enlarged and dorsally projected right prolongation of the tegmen with an apophysis on its dorsal face as synapomorphies of *Paramaronius*.

In this study, three new species of *Paramaronius* from southeastern Brazil are described. The genus now includes nine species totally, which are distributed from French Guiana to northern Argentina and from the southeastern Brazilian coast to Bolivia and western Brazil, most frequently on highlands.

## Material and methods

Acronyms of the institutions where the types and other examined specimens are deposited as following:

CEIOC Coleção Entomológica do Instituto Oswaldo Cruz, Rio de Janeiro, Brazil;

CZMA Coleção Zoológica do Maranhão, Caxias, Brazil;

DZUP Coleção de Entomologia Pe. Jesus Santiago Moure, Universidade Federal do Paraná, Curitiba, Brazil;

MNHN Muséum national d’Histoire naturelle, Paris, France;

MNRJ Museu Nacional do Rio de Janeiro, Rio de Janeiro, Brazil;

MZSP Museu de Zoologia da Universidade de São Paulo, São Paulo, Brazil;

NHM Natural History Museum, London, England;

NHMB Naturhistorisches Museum Basel, Basel, Switzerland.

The morphological terminology and methods of dissection follow Brancucci (1980, 1982) and Constantin (2008). Illustrations were produced via camera lucida attached to a Carl Zeiss Discovery V8 stereomicroscope. Photographs were taken with a Canon EOS Rebel T3i camera with Canon MP-E 65mm macro-lens, StackShot macro-rail and Griffi Equipamentos portable camera stand. Focus stacking was performed with software Zerene Stacker, version 1.04. Illustrations and photographs were edited in Adobe Photoshop CS6 and Adobe Illustrator CS6.

Localities for the distribution map were obtained from label data and compiled from literature (Wittmer 1963, Brancucci 1982, 1983). Those were plotted on software Google Earth v. 7.1.2.2041 and exported to Quantum GIS version 1.7.3-Wroclaw for edition of the final map, made with Natural Earth (2015).

Abbreviations of the genital structures: rpt: right prolongation of tegmen, ap: apophysis, lsp: left setiferous prolongation, rp: right paramere, lp: left paramere, ml: median lobe.

## Results

### Descriptions

#### Paramaronius serranus sp. n.

Taxon classificationAnimaliaColeopteraCantharidae

http://zoobank.org/042F416D-FD46-446B-8AD4-15ABF5738BB6

Figs 1–5 , 14 , 18 , 19 , 22 , 26 , 30

##### Type material.

HOLOTYPE ♂: BRAZIL: São Paulo, Jundiaí (Reserva Biológica Serra do Japi), 23°14'20"S; 46°57'27"W, 8.xi.2011, Biffi, G. & Nascimento E.A. cols. (MZSP). PARATYPES: BRAZIL: São Paulo, Jundiaí (Reserva Biológica Serra do Japi), 23°14'20"S; 46°57'27"W, 8.xi.2011, Biffi, G. & Nascimento E.A. cols. (2♂, 3♀) (MZSP); same locality, 23–25.i.2012, Nascimento, E.A., Biffi, G. & Fernandes, F.R. (1♀ pined; 1♂, 1♀ in alcohol) (MZSP); São Paulo, Bocaina [Serra da Bocaina], 27.x.1963, H.S. Lopes col. (1♂) (CEIOC); same locality, 27.x.1963, P. Ildo col. (1♀) (CEIOC); Minas Gerais (Serra do Caraça), 1380 m, xi.1961, Kloss, Lenko, Martins & Silva col. (1♂, 1♀) (MZSP).

##### Description.

Holotype (Figs 4, 5): Head with the occipital region, vertex and apex of mandibles dark brown; frons, clypeus, genae and base of mandibles pale yellow. Frons presents a small and barely defined brown spot between the antennae. Antennomeres 1-3 dark brown dorsally and pale yellow ventrally; antennomeres 4-11 dark brown on the apex and lighter on the base. Labial and maxillary palpi pale yellow, and last palpomeres, light brown dorsally. Pronotum dark brown, with two medial yellow spots: one round anteriorly and another sub-rectangular posteriorly, reaching the posterior margins; posterior margin, anterior angles and hypomera pale yellow. Scutellum yellow with translucent apex. Elytra dark brown with yellow apices. Hind wings dark brown. Thorax pale yellow ventrally, with anterior margins of meso- and metanepisterna brown. Legs pale yellow, with the base of coxae, dorsal face of femora, apical and dorsal faces of tibiae and tarsi, brown. Abdominal tergites dark brown, with yellow borders; posterior borders of tergites VI and VII yellow; ventrites yellow with brown spots laterally; two last ventrites brown.

**Figures 1–5.**
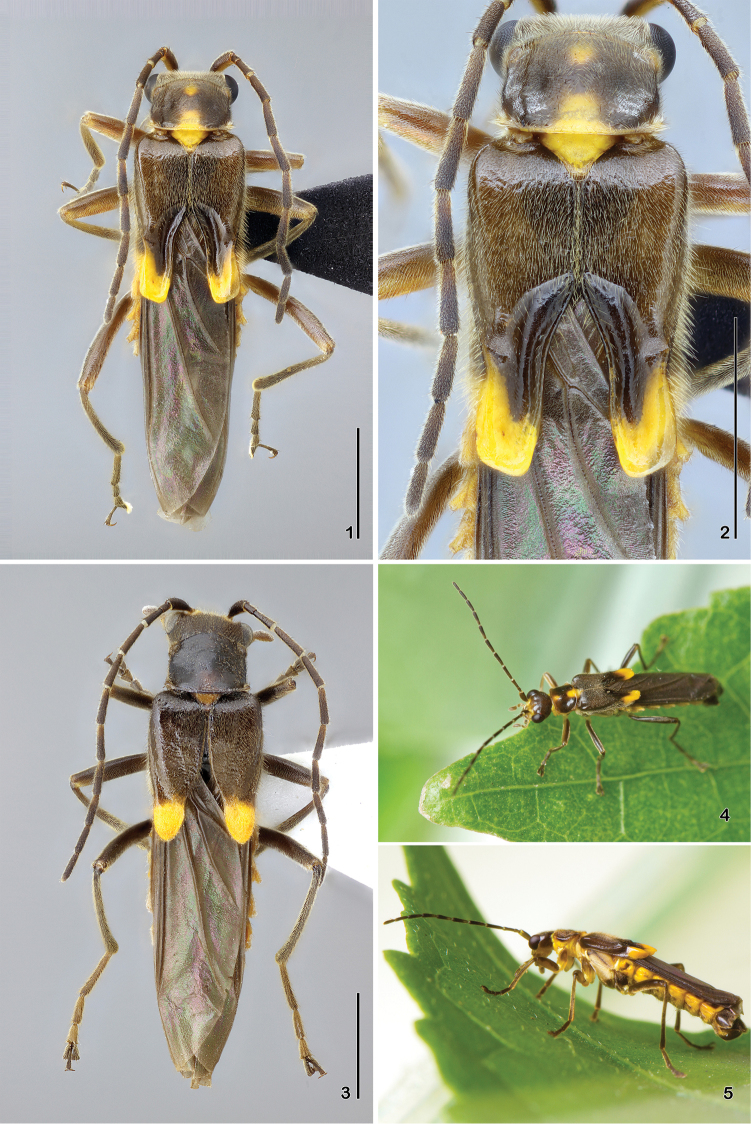
*Paramaronius serranus* sp. n. **1** male habitus, dorsal view **2** male elytra, dorsal view **3** female habitus, dorsal view **4** live male habitus, dorsal view **5** live male habitus, lateral view. Scale bars: 2.0 mm.

Male (Fig. 1): Head covered with fine and dense pubescence; vertex and occipital region flat; lateral margin of head arcuated behind the eyes. Eyes prominent. Antennae long, last two antennomeres exceeding the apex of elytra; antennomere 1 slender, 3.6 times longer than wide; antennomere 2 short, third antennomere 2 times shorter than antennomere 1, fourth to sixth subequal in length, as long as the antennomere 1, seventh antennomere is the longest, slightly longer than the antennomere 1, eighth to eleventh antennomeres progressively shorter than the seventh. Pronotum 1.15 times wider than long, longer at middle; anterior and posterior margins arcuated; lateral margins slightly sinuate; posterior angles directed upwards. Scutellum wide, triangular, with truncate apex. Elytra short, 1.5 times longer than wide; pubescence short, fine and dense; sutures dehiscent from apical half; apical half modified into a shallow impression forming glabrous slopes; truncate apex. Epipleura with a row of thin and longer setae born on a shallow groove directed backwards to meet a slight fold on dorsal face (Fig. 2). Legs slender, hind tibia longer than hind femur; tarsomeres gradually increasing in size from fore to hind legs; first metatarsomere 1.9 times longer than second and 2.6 times longer than third. Abdominal glandular pores slightly prominent. Seventh abdominal ventrite (Fig. 14) wider than long, deeply emarginated forming two sharpened lobes with membranous apices. Aedeagus (Fig. 22) with right prolongation of tegmen large and slightly sinuous on the posterior margin, covered by long setae. Dorsal surface with a short apophysis. Left setiferous prolongation very long and curved, not clubbed, with few setae at apex. Right paramere (Fig. 26) short, narrow at base and very wide at apex, directed backwards, meeting dorsal apophysis of tegmen. Left paramere flat, short and wide, divided apically and partially covering the dorsal surface of median lobe. Median lobe (Fig. 30) long and curved to the right. Ventral and lateral surfaces of tegmen smooth.

Female (Fig. 3) slightly bigger than male; eyes smaller and less prominent; pronotum longer than of male with anterior margin more arched and lateral margins almost straight; dorsal surface of elytra without sculptural modifications; sutures gradually dehiscent from apical half. Seventh abdominal ventrite (Fig. 18) trapezoidal, with distal margin sinuate and emarginated at middle. Coxites elongated, narrow basally and more sclerotised at distal margins. Styles (Fig. 20) long and straight.

##### Color variations.

Other specimens from the type locality are much more pigmented. In these cases the frons, clypeus, palpi, thorax and legs vary from dark brown to black; the yellow pronotal spots may be indistinct. On less pigmented specimens, the head is pale with a posterior V-shaped mark; pronotum broadly pale, the sides light brown; elytra light brown, with the apex yellow.

##### Etymology.

The specific epithet is a derivative of the Portuguese word “serra” (mountain ranges), referring to the regions where the specimens were collected.

##### Distribution.

Brazil (Minas Gerais and São Paulo) (Figs 44, 45).

##### Biological data.

The specimens from Serra do Japi were collected on shrub vegetation across the most humid part of the trail near the Paraíso stream. The local vegetation is composed of a highland mesophyllous semideciduous forest (Leitão-Filho 1992).

#### Paramaronius brancuccii sp. n.

Taxon classificationAnimaliaColeopteraCantharidae

http://zoobank.org/8418A273-638E-46D8-A947-4C676ABBDA6B

**Figures 6–9.**
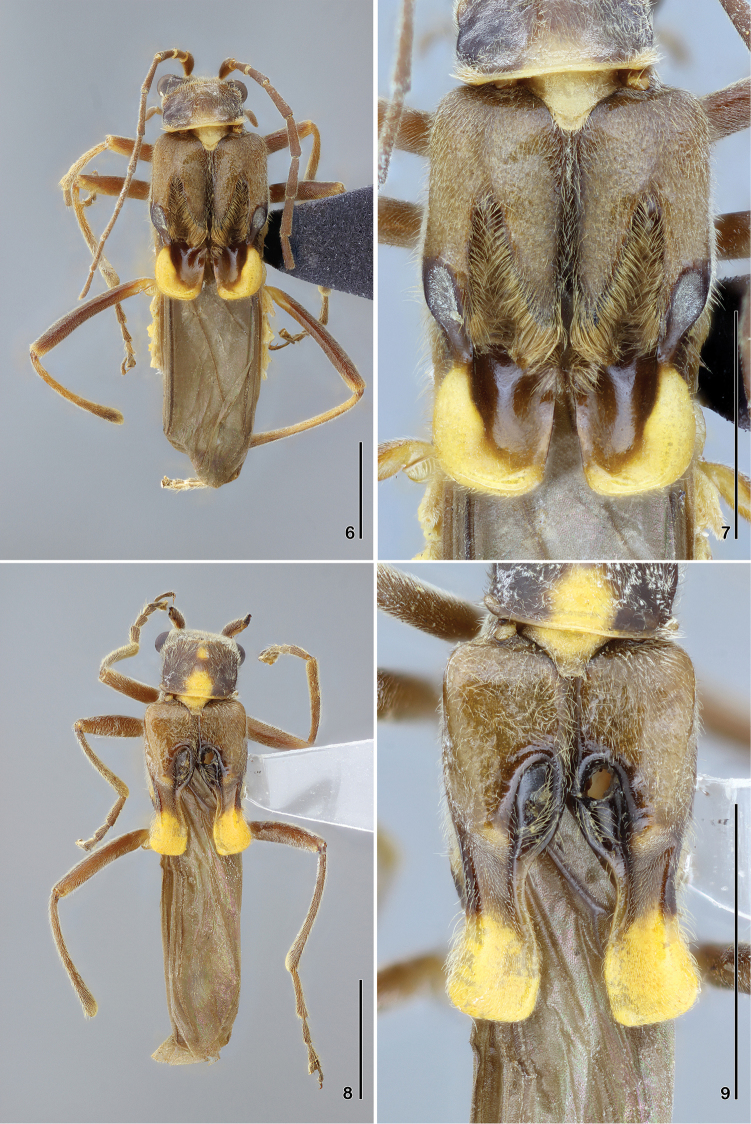
**6–7** *Paramaronius brancuccii* sp. n. **6** male habitus, dorsal view **7** male elytra, dorsal view **8–9** *Paramaronius cavipennis* sp. n. **8** male habitus, dorsal view **9** male elytra, dorsal view. Scale bars: 2.0 mm.

Figs 6 , 7 , 15 , 23 , 27 , 31

##### Type material.

HOLOTYPE ♂: BRAZIL: São Paulo, Pindamonhangaba (Eugênio Lefévre), 26.x.1962, Exp. Dep. Zool. col. (MZSP). PARATYPES: BRAZIL: São Paulo, Pindamonhangaba (Eugênio Lefévre), 26.x.1962, Exp. Dep. Zool. col. (1♂) (MZSP); Minas Gerais, Monte Verde, 10.xii.1969, F. Halik. (9266) (1♂) (MZSP)

##### Description.

Head, clypeus and apex of mandibles dark brown; antennal sockets surrounded by a thin yellow ring; base of mandibles and labial and maxillary palpi light brown; last palpomeres darker. Antennae dark brown, with the ventral face of antennomere 1 lighter. Pronotum dark brown laterally and light brown medially; a barely defined yellow spot near anterior medial margin and a broader, yellow, medial, basal spot reaching the posterior margin; posterior angles and hypomera pale yellow. Scutellum pale yellow. Elytra dark brown, with yellow apex. Hind wings brown. Thorax pale ventrally; metathorax gradually darker posteriorly. Legs dark brown, with apex of coxae and ventral surface of femora, pale. Abdominal tergites dark brown, with lateral borders yellow; posterior borders of tergites VI and VII yellow; ventrites dark brown with yellow spots basally and laterally; two last ventrites light brown.

Male (Fig. 6): Head covered with fine and dense pubescence; vertex and occipital region flat; lateral margin of head arcuate behind eyes. Eyes prominent. Antennae slender and long, last two antennomeres exceeding the apex of elytra; antennomere 1 slender, 3.6 times longer than wide; antennomere 2 short, third antennomere slightly shorter than antennomere 1, fourth to ninth antennomeres as long as the antennomere 1, tenth and eleventh slightly shorter than antennomere 1. Pronotum 1.1 times wider than long; anterior and posterior margins slightly arcuate; lateral margins slightly sinuate; posterior angles directed upwards. Scutellum wide, triangular, apex slightly rounded. Elytra (Fig. 7) short, 1.4 times longer than wide; pubescence long and dense; sutures slightly dehiscent apically; dorsal surface modified into a deep longitudinal incision which widen apically and are covered by a dense, long and thick pubescence. External margin of each elytron constricted laterally and with a large tubercle; apices broadly rounded. Legs slender; hind tibia longer than hind femur; tarsomeres gradually increasing in size from fore to hind legs; first metatarsomere twice longer than second and 3 times longer than third. Abdominal glandular pores slightly prominent. Seventh abdominal ventrite (Fig. 15) wider than long and deeply emarginated, forming two apical, narrow lobes with margins truncate. Aedeagus (Fig. 23) with right prolongation of tegmen very large and sinuous on posterior margin, with angles rounded and prominent laterally, and covered by large setae. Dorsal surface with a short apophysis. Left setiferous prolongation (Fig. 23) long and curved, slightly clubbed, with few setae at apex. Right paramere (Fig. 27) long, narrow at the base and divided in two asymmetrical processes: one lateral, short and one longitudinal, long, which reaches the apophysis of tegmen. Left paramere flat, short and wide, divided apically and partially covering dorsal surface of median lobe. Median lobe short and slightly curved to the right. Ventral and lateral surfaces of tegmen smooth (Fig. 31).

Female: Unknown.

##### Etymology.

The specific epithet is patronymic, in honor of Dr Michel Brancucci (NHMB), eminent Cantharidae taxonomist, especially devoted to the Chauliognathinae, deceased in 2012 (Klausnitzer 2012).

##### Distribution.

Brazil (Minas Gerais and São Paulo) (Figs 44, 45).

#### Paramaronius cavipennis sp. n.

Taxon classificationAnimaliaColeopteraCantharidae

http://zoobank.org/7E1948A7-66DB-4CB1-9DC0-A96BBB150975

Figs 8 , 9 , 16 , 24 , 28 , 32

##### Type material.

HOLOTYPE ♂: BRAZIL: Minas Gerais, Serra do Caraça, Santa Bárbara, 23–25.xi.1960, Araujo & Martins col. (MZSP).

##### Description.

Head with the occipital region and vertex dark brown; frons, clypeus and mandibles light brown; base of antennae, genae, labium and ventral base of mandibles yellow. Antennae with antennomere 1 dark brown dorsally and light brown ventrally; last antennomeres lost. Labial and maxillary palpi light brown. Pronotum dark brown, with a median anterior small round yellow spot and a basal, median, bigger, yellow spot reaching posterior margin; hypomera yellow. Scutellum pale yellow with the apex translucent. Elytra light brown with yellow apices. Hind wings brown. Ventrally, thorax light brown, with anterior margins of meso- and metanepisterna dark brown. Legs totally dark brown. Abdominal tergites light brown with lateral margins yellow; ventrites light brown, with lateral margins yellow covered with dark brown spots; two last tergites and ventrites dark brown.

Male (Fig. 8): Head with short and dense pubescence; vertex and occipital region flat; lateral margins of head arcuate behind eyes. Antennae with antennomere 1 slender, 2.7 times longer than wide. Pronotum 1.1 times wider than long; anterior and posterior margins slightly rounded; lateral margins slightly sinuate; posterior angles slightly directed upwards. Scutellum wide, triangular, apex truncate. Elytra (Fig. 9) short, 1.5 times longer than wide; dorsal surface modified in a deep hollow surrounded by a scarce, long and thin pubescence, followed by a sloped surface covered by very short pubescence; external margins of elytra constricted laterally; sutures abruptly dehiscent from apical half; apex truncate, with rounded angles. Legs slender; hind tibia slightly longer than hind femur; tarsomeres gradually increasing in size from fore to hind legs; first metatarsomere 1.15 times longer than second and 2.5 times longer than third. Abdominal glandular pores slightly prominent. Seventh abdominal ventrite (Fig. 16) wider than long, densely pubescent, deeply emarginated forming two apical wide lobes with truncate apices. Aedeagus (Fig. 24) with a large right prolongation of tegmen, slightly sinuous on posterior margin, with one angle well developed and other truncate, and rather straight on lateral margin, covered with large setae. Dorsal surface with projecting apophysis. Left setiferous prolongation (Figs 24, 32) long and curved, slightly clubbed, with few setae at apex and on the dorsal surface. Right paramere (Fig. 28) long, curved, narrowing upwards and divided in two opposite processes reaching apophysis of tegmen. Left paramere flat, short and wide, divided apically and covering the base of dorsal surface of median lobe. Median lobe (Fig. 32) long and slightly curved to the right. Ventral and lateral surfaces of tegmen smooth.

Female: Unknown.

##### Etymology.

The specific epithet is derivative of the Latin words *cavum* (cavity) + *pennis* (wings), referring to its deep hollows on dorsal surface of elytra.

##### Distribution.

Brazil (Minas Gerais) (Fig. 44, 45).

#### Paramaronius impressipennis

Taxon classificationAnimaliaColeopteraCantharidae

(Pic, 1906)

Figs 10–13 , 17 , 19 , 21 , 25 , 29 , 33

##### Remarks.

This species was briefly described by Pic (1906) based on a male from Tucumán, northern Argentina. However, some specimens have been collected in the northeastern region of Brazil (states of Bahia and Maranhão), far away from the type locality. The comparison of the Brazilian specimens with the holotype (MNHN) shows there are no observable differences within them and they are, indeed, the same species.

Even though these localities are so distant, they belong to Cerrado and Chaco provinces, adjacent areas of the same biogeographic subregion (Chaco dominion) (Morrone 2000, 2006) (Fig. 44).

A redescription of *Paramaronius impressipennis* is presented as well as a supplementary description of the female for the first time to complete its morphological information after specimens from Brazil.

##### Material examined.

HOLOTYPE ♂ (MNHN): ARGENTINA: Tucumán, 4.i.1900. BRAZIL: Maranhão, Mirador (Parque Estadual do Mirador, Base da Geraldina) 6°46'37"S; 45°06'34"W, 22.ii–01.iii.2009, armadilha luminosa [light trap], F. Limeira-de-Oliveira col. (4♂, 14♀ MZSP, 4♂, 15♀ CZMA); same locality, (Parque Estadual do Mirador, Base do Mosquito), 04–08.ii.2011, armadilha luminosa, F. Limeira-de-Oliveira col. (1♀) (CZMA); Bahia, Barreiras (Estrada Mata de Cachoeiras, Acaba Vida, km 7), 11°52'20"S; 45°32'55"W, 23.i.2009, luz [light trap], Nihei, Figueiredo, Almeida & Cezar col. (1♀) (MZSP).

##### Redescription.

Head with vertex, frons, clypeus and bases of mandibles pale yellow, slightly translucent; apex of mandibles dark brown; occipital region with a V-shaped testaceous mark; labial and maxillary palpi pale yellow, last palpomere brown. Antennae light brown, three first antennomeres pale yellow. Pronotum pale yellow to light brown, sometimes translucent. Scutellum pale yellow with apex translucent. Elytra testaceous to light brown with a lateral longitudinal darker brown band from the epipleura to the posterior third quarter of dorsal surface; apex of elytra with an oblique yellow band on outer margin; on less pigmented specimens, pronotum pale yellow and lateral and apical patches indistinct. Hind wings light brown. Legs testaceous to light brown; tarsi and dorsal surface of tibiae darker. Ventrally, thorax and abdomen pale yellow.

**Figures 10–13.**
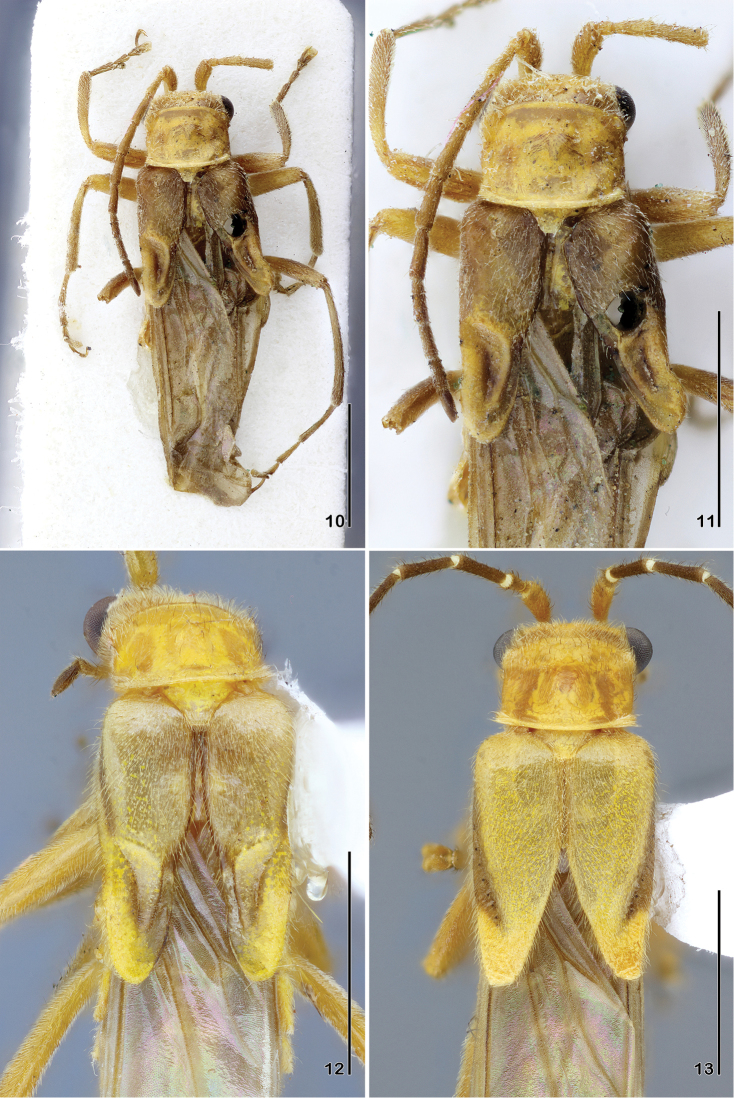
**10–11** *Paramaronius impressipennis* (Pic), holotype **10** habitus, dorsal view **11** elytra, dorsal view **12–13** *Paramaronius impressipennis* (Pic) from Brazil **12** male elytra, dorsal view **13** female elytra, dorsal view. Scale bars: 2.0 mm.

Male (Fig. 10): body covered by dense and fine pubescence. Lateral margins of head arcuate behind eyes; vertex slightly convex, frons flat. Eyes prominent. Antennae short, with one antennomere exceeding the apex of elytra. Antennomere 1 long and swollen, 2.7 times longer than wide; antennomere 2 short, third antennomere 1.7 times shorter than antennomere 1, forth to seventh subequal in length, slightly shorter than antennomere 1, the latter progressively shorter than seventh. Pronotum subrectangular, shiny, slightly narrower near fore angles; anterior margin slightly rounded. Scutellum triangular, apex truncate. Elytra short, 1.5 times longer than wide, covered by fine hairs and some sparse black hairs. Apical half modified, forming a longitudinal ridge and two sloped surfaces covered by very short hairs (Figs 11, 12). Legs slender; hind tibia as long as hind femur; tarsomeres gradually increasing in size from fore to hind legs; first metatarsomere 1.7 times longer than second and 2.7 times longer than third. Abdominal glandular pores slightly prominent. Seventh abdominal ventrite (Fig. 17) wider than long, deeply emarginate, forming two distal lobes, narrowed apicad. Aedeagus (Fig. 25) with right prolongation of tegmen large and arcuate on posterior margin, and covered with large setae; dorsal surface with a short and rounded apophysis; left setiferous prolongation very long and curved, clubbed apically, with few setae on its apex. Right paramere (Fig. 29) short, narrowing apicad, then divided at apex, forming an inclined prolongation, reaching apophysis of tegmen; left paramere flat, very short and wide, divided apically and partially covering dorsal surface of median lobe; median lobe long and curved to right, sometimes retracted. Ventral and lateral surfaces of tegmen (Fig. 33) with fine scratches.

**Figures 14–21.**
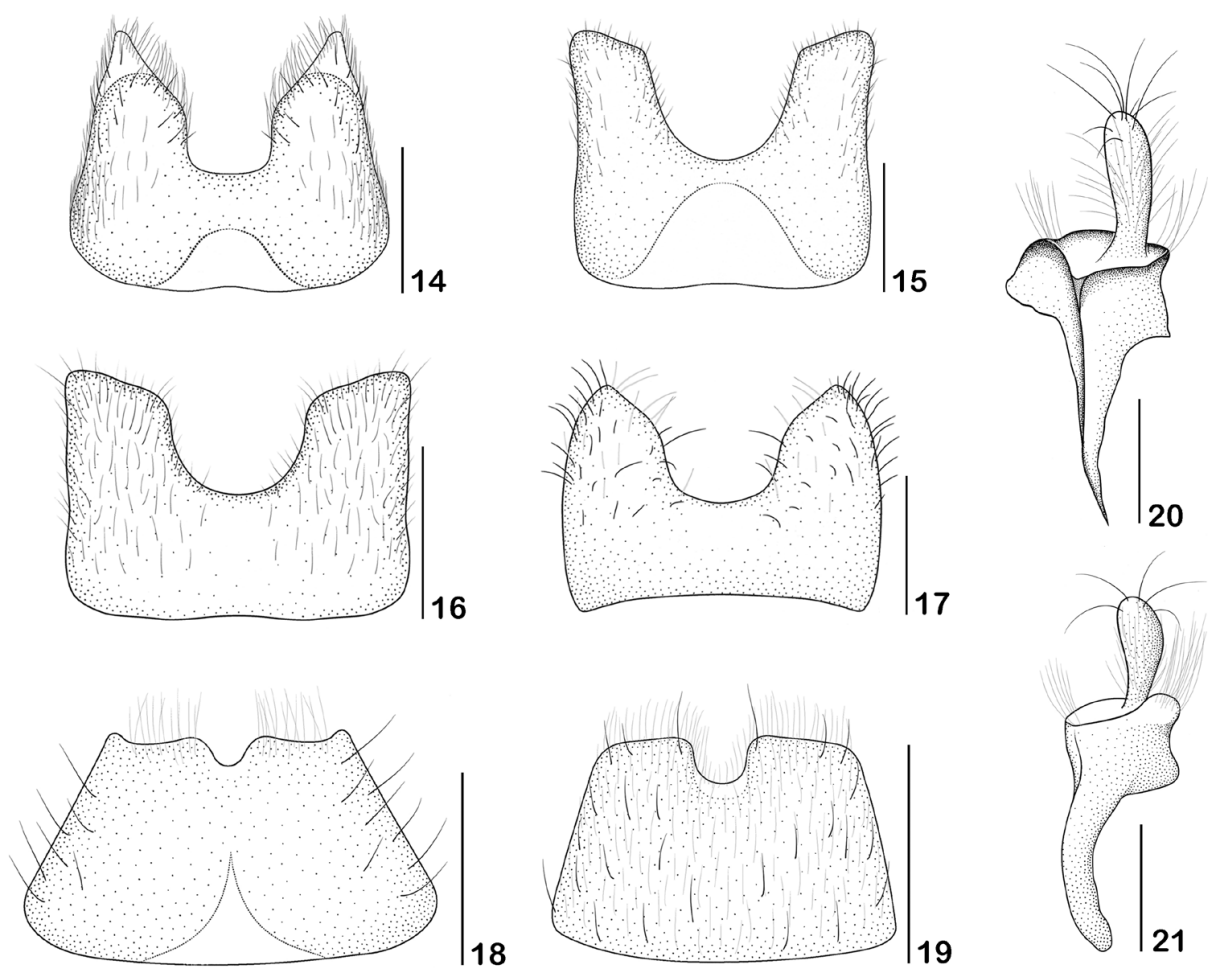
**14–17** Seventh abdominal ventrite of male, ventral view **18–19** seventh abdominal ventrite of female, ventral view **20–21** female right coxite, ventral view **14, 18, 20** *Paramaronius serranus* sp. n. **15** *Paramaronius brancuccii* sp. n. **16** *Paramaronius cavipennis* sp. n. **17, 19, 21** *Paramaronius impressipennis* (Pic). Scale bars: 0.5 mm (**14–19**); 0.2 mm (**20–21**).

**Figures 22–29.**
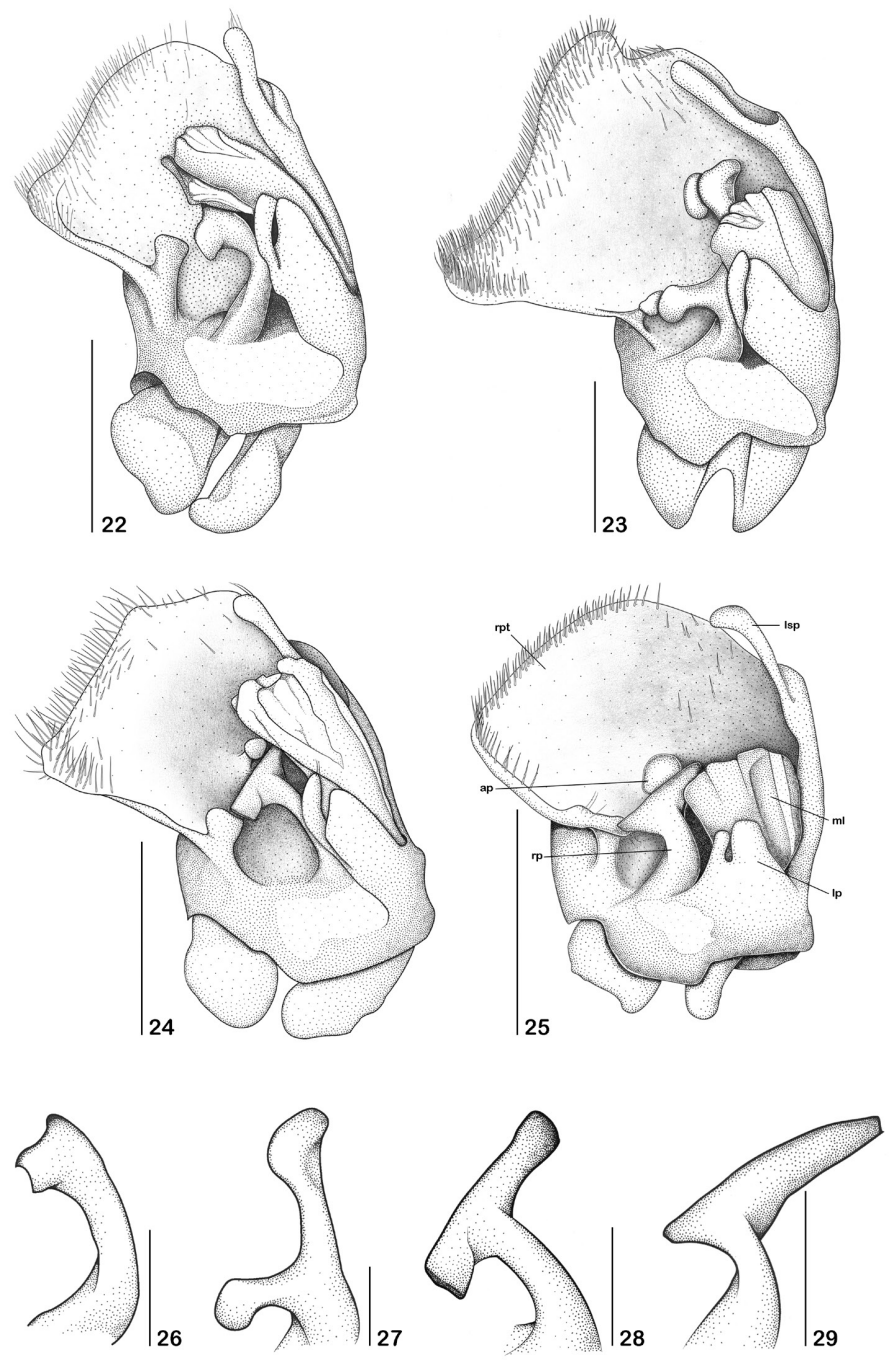
**22–25** Aedeagus, dorsal view **26–30** right paramere, dorsal view **22, 26** *Paramaronius serranus* sp. n. **23, 27** *Paramaronius brancuccii* sp. n. **24, 28** *Paramaronius cavipennis* sp. n. **25, 30** *Paramaronius impressipennis* (Pic). Scale bars: 0.5 mm (**22–25**); 0.2 mm (**26–29**).

**Figures 30–33.**
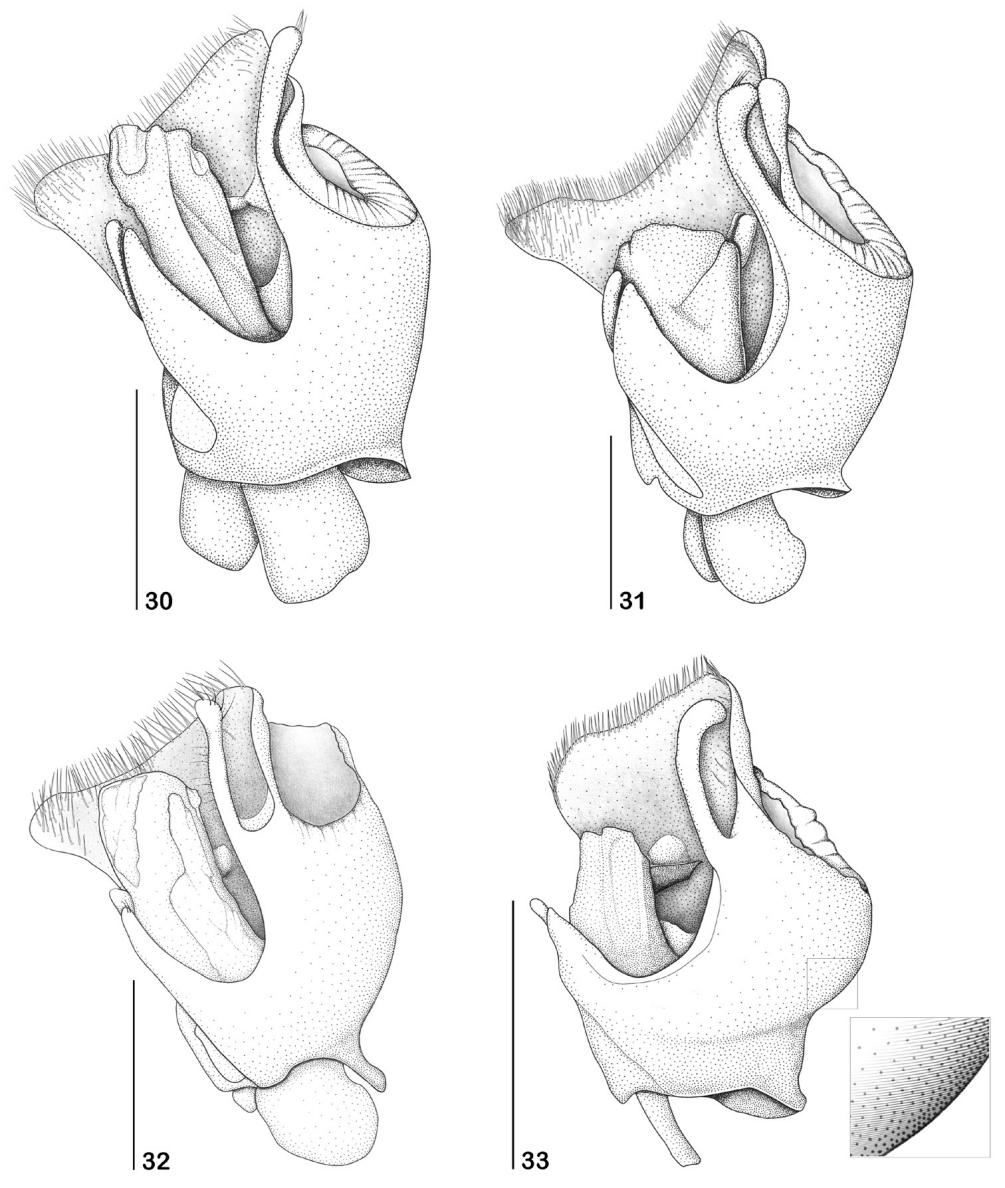
Aedeagus, left view **30** *Paramaronius serranus* sp. n. **31** *Paramaronius brancuccii* sp. n. **32** *Paramaronius cavipennis* sp. n. **33** *Paramaronius impressipennis* (Pic). Scale bars: 0.5 mm.

Female (Fig. 13) slightly bigger than male; antennomere 1 slender, not swollen; dorsal surface of elytra without sculptural modification, brown lateral and yellow apical bands more distinct. Seventh abdominal ventrite (Fig. 19) trapezoidal, distal margin straight with a short, rounded notch. Coxites (Fig. 21) small and membranous; styles short, wider apically.

##### Distribution.

Argentina (Tucumán) and Brazil (Maranhão and Bahia) (Fig. 44).

##### Biological data.

The specimens from Maranhão and Bahia were collected on Brazilian savannah (cerrado) and were attracted by light traps.

#### Paramaronius kraatzi

Taxon classificationAnimaliaColeopteraCantharidae

(Pic, 1938)

Figs 34 , 35

##### Material examined.

BOLIVIA: Cochabamba, Chaparé, Locotal, 1200m, 8.xi.1953, Martinez leg. (4♂); Cochabamba, Chaparé, Yungas de Palmar, 1250m 17.x.1953, W. Foster (1♀) (NHMB); Santa Cruz, Florida, 1050-1150m, (Refugio Los Volcanes), 18°06,3'S; 63°26'W, 10-14.xii.2011, beating of vegetation, L. Sekerka lgt. (2♀) (NHM).

##### Distribution.

Bolivia (Cochabamba and Santa Cruz) (Fig. 44).

#### Paramaronius freyi

Taxon classificationAnimaliaColeopteraCantharidae

Wittmer, 1963

Figs 36 , 37

##### Remarks.

This species was described based on a single male holotype from northwestern Brazil, labelled “Brasilien, Acre, Rio Branco, 29.x.1954” and preserved at NHMB. Several specimens were collected with interception traps disposed for faunistic inventory purpose at Saül, French Guiana, by the SEAG (Société entomologique Antilles-Guyane). The specimens from French Guiana were compared and found to be identical to the holotype.

##### Material examined.

FRENCH GUIANA, Saül, Belvédère de la Montagne Pelée, 320m, 3°37'22"N; 53°12'58"W, 20.xii.2010, P.H. Dalens & S. Brûlé (1♀) (MZSP), same locality, 320m, 3°37'22"N; 53°12'58"W, 24.i.2011, P.H. Dalens & S. Brûlé, SEAG (1♂) (MZSP).

##### Distribution.

French Guiana and Brazil (Acre) (Fig. 44).

#### Paramaronius gounellei

Taxon classificationAnimaliaColeopteraCantharidae

(Pic, 1906)

Figs 38 , 39

##### Material examined.

BRAZIL: Minas Gerais, Serra do Caraça, 1380m, xi.1961, Kloss, Lenko, Martins & Silva col. (2♂, 6♀) (MZSP); Serra do Caraça (Engenho), 800m, xi.1961, Kloss, Lenko, Martins & Silva col. (1♂) (MZSP); Serra do Caraça, Santa Bárbara, 23–25.xi.1960, Araujo e Martins col. (2♀) (MZSP); Minas Gerais, Poços de Caldas (morro de São Domingos), 12.ii.1969, J. Becker, O. Roppo & O. Leoncini cols. (1♀) (MNRJ); Rio de Janeiro, Serra de Macaé, xi.1909, E. Garbe (1♂) (MZSP 15235); Parque Nacional de Itatiaia, 10.xii.1950, L. & H. Travassos (1♂) (MNRJ); Rio de Janeiro, Itatiaia, xi.1950, Travassos & Dalcy, (1♂) (MNRJ); Rio de Janeiro, Itatiaia (L. 41, 1300m), 10–12.x.1950, Trav., Albuquerque & Pearson col. (1♂) (CEIOC); same locality, 6–10.x.1950, H. Trav. col. (1♀) (CEIOC); Rio de Janeiro, Teresópolis, i.1940, Trav. & Freitas col. (2♀) (CEIOC); São Paulo, Serra da Bocaina, São José do Barreiro, 1650m, i.1969, M. Alvarenga col. (2♂) (DZUP 273518; DZUP 273519); São Paulo, Serra da Bocaina, 1300m, Parq. Criaç. Trutas, iii.1954, Dalcy, R. Barros (1♀) (MNRJ); São Paulo, Salesópolis, Estação Biológica de Boracéia, 23°39'15"S; 45°53'22"W, light trap, 14-18.ix.2012, F.F. Albertoni col. (1♂) (MZSP).

##### Distribution.

Brazil (Minas Gerais, Rio de Janeiro and São Paulo) (Fig. 44, 45).

##### Note.

One female referred by Brancucci (1982) as “BRAZIL: Mato Grosso do Sul, Corumbá, Serra do Urucum” (MZSP) in unlikely a *Paramaronius gounellei*. This damaged specimen cannot be precisely identified and is referred to the distribution map as *Paramaronius* sp. (Fig. 44).

#### Paramaronius campbelli

Taxon classificationAnimaliaColeopteraCantharidae

Brancucci, 1983

Figs 40 , 41

##### Material examined.

HOLOTYPE ♂ (MZSP) and 1 PARATYPE ♂ (NHMB): BRAZIL: Distrito Federal, Parque Nacional, 1000 m, 9.iii.1970, JM & BA Campbell; 1 PARATYPE ♀ (NHMB): Distrito Federal, 15 km N. Brasília, 1250 m, 5.iii.1970, JM & BA Campbell. Other material: BRAZIL: Goiás, Ribeirão Vãozinho, 12.ii.1962, J. Bechyné col. (1♂, 2♀) (MZSP).

##### Distribution.

Brazil (Goiás and Distrito Federal) (Figs 44, 45).

#### Paramaronius menieri

Taxon classificationAnimaliaColeopteraCantharidae

Brancucci, 1982

Figs 42 , 43

##### Material examined.

HOLOTYPE ♂ (MNHN): BRAZIL, Goiás, Jataí, coll. L. Fairmaire, 1906.

##### Distribution.

Brazil (Goiás) (Figs 44, 45).

### Distribution of *Paramaronius*

*Paramaronius* is widely distributed throughout South America. The new records extend the distribution of the genus from French Guiana to northern Argentina (Tucumán province) and from the southeastern Brazilian coast (Rio de Janeiro and São Paulo states) to western Brazil (Acre state) and Bolivia (Cochabamba department) (Figs 44, 45). The species are distributed through a wide variety of biomes and vegetation, most frequently present on mountains at mid altitude (1000 m to 1700 m): *Paramaronius gounellei*, *Paramaronius serranus* sp. n., *Paramaronius brancuccii* sp. n. and *Paramaronius cavipennis* sp. n. occur on the southeastern South American Atlantic Forest (Fig. 45); *Paramaronius campbelli*, *Paramaronius menieri* and *Paramaronius impressipennis* are present in the Brazilian savannah (cerrado) of the great plateau of central Brazil. *Paramaronius freyi* occurs in dense rainforests in northern and western Amazon, while *Paramaronius kraatzi* occurs in the Bolivian Yungas, a transition area between the Amazon and the highlands (Fig. 44). However, the discussion of distribution patterns of the species is still unfeasible due to the scarce records for species of *Paramaronius*.

### Identification Key

**Table:** 

1	Antennae short, with one antennomere exceeding or barely reaching the apex of elytra; males: elytra with one or two longitudinal ridges on posterior half of dorsal surface; sides of tegmen finely scratched	**2**
–	Antennae long, with two or more antennomeres exceeding the apex of elytra; males: elytra with strong tubercles, deep grooves or shallow impressions on internal margins; sides of tegmen tuberculated or smooth, never scratched	3
2	Head black; males: antennomeres 6–11 expanded; elytra with two ridges on the posterior half and truncate to slightly emarginate apex (Figs 42, 43)	***Paramaronius menieri* Brancucci**
–	Head yellow; males: antennomeres not expanded; elytra with one ridge on the posterior half and apex rounded (Figs 10–13)	***Paramaronius impressipennis* (Pic)**
3	Legs completely or mostly testaceous, sometimes light brown; males: elytra widely rounded on posterior margins and provided with strong tubercles; sides of tegmen tuberculated	**4**
–	Legs completely or mostly dark brown to black; males: elytra truncate or slightly rounded on posterior margins and without strong tubercles; sides of tegmen smooth	**5**
4	Head, pronotum and legs completely testaceous; antennae testaceous, darker from antennomere 7 to apex; males: each elytron with one median and two apical tubercles (Figs 34, 35)	***Paramaronius kraatzi* (Pic)**
–	Head black; pronotum completely yellow or with two brown spots on lateral margins; legs yellow with brown spots on apex of femora and tibiae, tarsi brown; antennae brown, slightly lighter on two first and three last antennomeres; males: each elytron with one median and three apical tubercles (Figs 36, 37)	***Paramaronius freyi***
5	Pronotum mostly or completely pale yellow; males: dorsal surface of elytra with shallow impressions covered by strong setae on their anterior margins	**6**
–	Pronotum mostly or completely dark brown to black; males: dorsal surface of elytra with shallow impressions not covered by strong setae or with deep longitudinal grooves	**7**
6	Head yellow, sometimes with two brown spots between the eyes and a V-shaped brown spot on occipital region; pronotum yellow with brown spots on sides; elytral suture yellow at apical third; males: dorsal surface of elytra with a straight shallow impression (Figs 38, 39)	***Paramaronius gounellei* (Pic)**
–	Head dark brown on vertex and yellow on clypeus and frons; pronotum completely testaceous to almost completely brown; elytral suture entirely black; males: dorsal surface of elytra with a circular impression (Figs 40, 41)	***Paramaronius campbelli* Brancucci**
7	Males: elytra with shallow impressions on internal margins not covered by dense pubescence (Figs 1–5)	***Paramaronius serranus* sp. n.**
–	Males: elytra with deep hollows or grooves surrounded by pubescence	**8**
8	Males: dorsal surface of each elytron modified in a deep longitudinal incision covered by a dense, short and thick pubescence; apical half of internal margin of each elytron slightly arcuate (Figs 6, 7)	***Paramaronius brancuccii* sp. n.**
–	Males: dorsal surface of each elytron modified in a deep hollow surrounded by a scarce, long and thin pubescence, followed by a sloped surface covered by very short pubescence; apical half of internal margin of each elytron deeply sinuate (Figs 8, 9)	***Paramaronius cavipennis* sp. n.**

**Figures 34–37.**
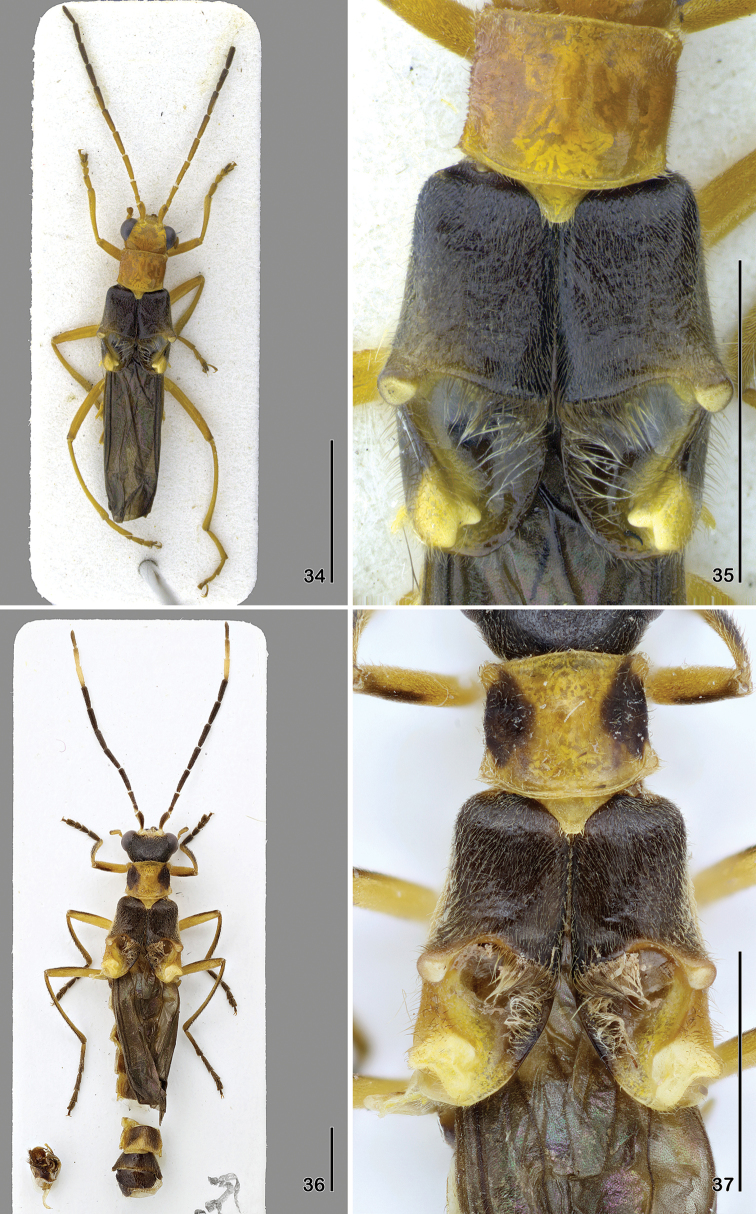
**34–35** *Paramaronius kraatzi* (Pic) **34** male habitus, dorsal view **35** male elytra, dorsal view **36–37** *Paramaronius freyi* Wittmer **36** male habitus, dorsal view **37** male elytra, dorsal view. Scale bars: 2.0 mm.

**Figures 38–41.**
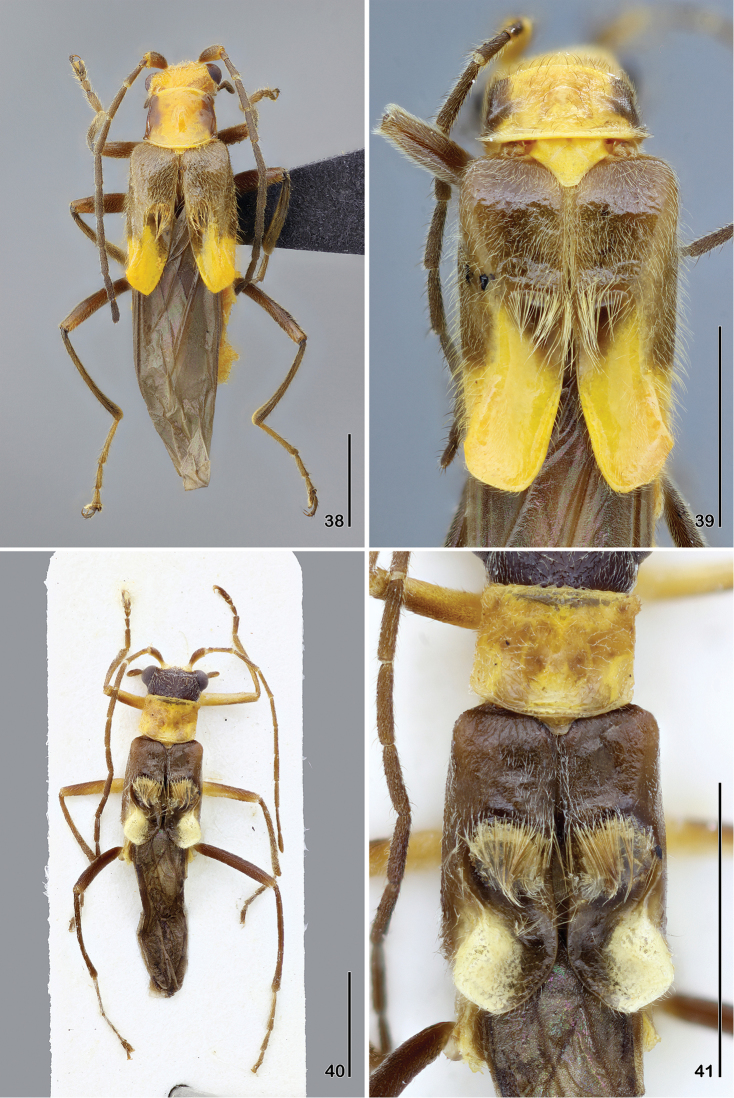
**38–39** *Paramaronius gounellei* (Pic) **38** male habitus, dorsal view **39** male elytra, dorsal view **40–41** *Paramaronius campbelli* Brancucci, holotype **40** male habitus, dorsal view **41** male elytra, dorsal view. Scale bars: 2.0 mm.

**Figures 42–43.**
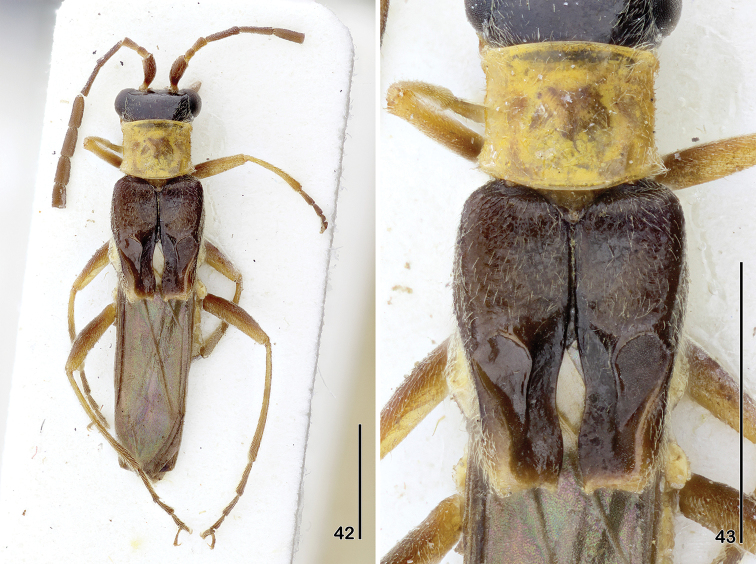
*Paramaronius menieri* Brancucci, holotype **42** male habitus, dorsal view **43** male elytra, dorsal view. Scale bars: 2.0 mm.

**Figure 44–45.**
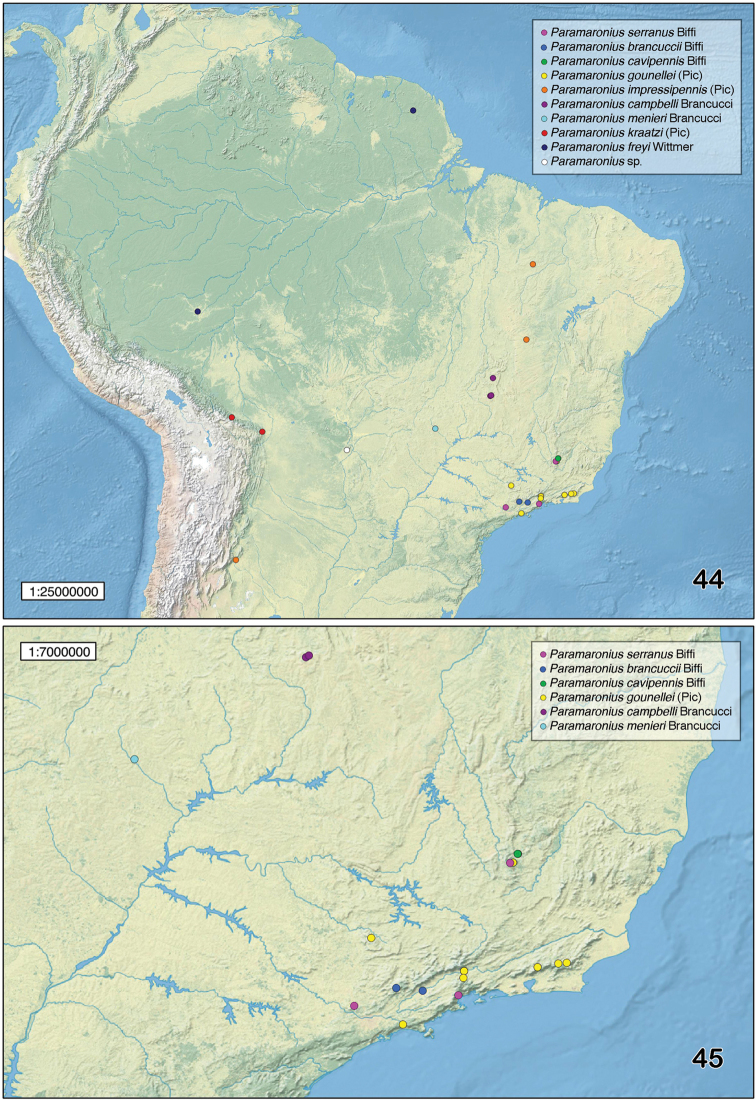
Distribution records for *Paramaronius* Wittmer.

## Supplementary Material

XML Treatment for Paramaronius serranus

XML Treatment for Paramaronius brancuccii

XML Treatment for Paramaronius cavipennis

XML Treatment for Paramaronius impressipennis

XML Treatment for Paramaronius kraatzi

XML Treatment for Paramaronius freyi

XML Treatment for Paramaronius gounellei

XML Treatment for Paramaronius campbelli

XML Treatment for Paramaronius menieri
